# Supplementary figures and images for: Giant flagellins form thick flagellar filaments in two species of marine γ-proteobacteria
Source: PLoS One. 2018 Nov 21;13(11):e0206544. doi: 10.1371/journal.pone.0206544 (PMC6248924; doi:10.1371/journal.pone.0206544)

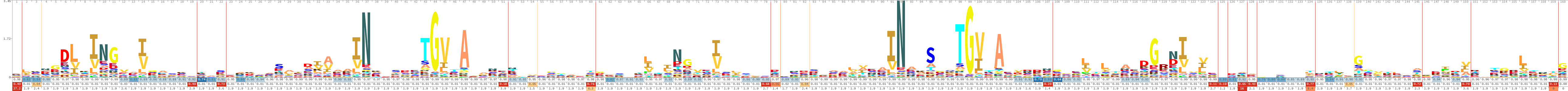

Supplement: S1 Fig — (PNG) [file pone.0206544.s009.png]
