# Supplementary material for: Giant flagellins form thick flagellar filaments in two species of marine γ-proteobacteria
Source: PLoS One. 2018 Nov 21;13(11):e0206544. doi: 10.1371/journal.pone.0206544 (PMC6248924; doi:10.1371/journal.pone.0206544)
Supplement: S2 Fig — (A) Overview of the whole alignment. (B) Detailed view of the alignment at the flagellin region (magenta box) showing 100% local identity. (C) Detailed view of the only sequence disagreement: an 84 bp intergenic region (olive box) present in the reference but not our assembly. The giant flagellin gene is represented by a magenta arrow. Other coding sequences are represented by yellow arrows. (PDF) [file pone.0206544.s010.pdf]

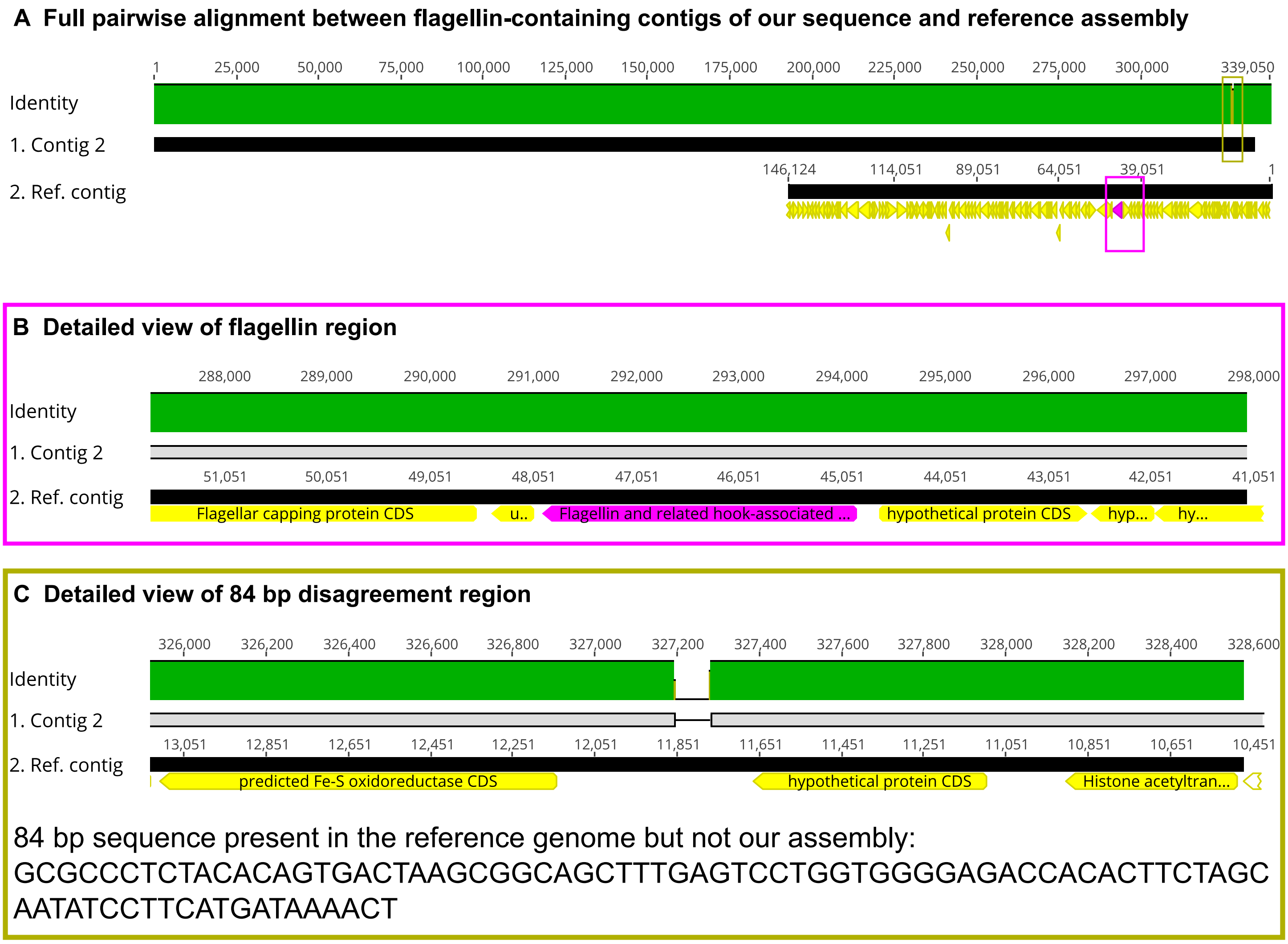

S2 Fig. Pairwise alignment of the flagellin-containing contigs in our *B. marisrubri* genome assembly and the reference assembly. (A) Overview of the whole alignment. (B) Detailed view of the alignment at the flagellin region (magenta box) showing 100% local identity. (C) Detailed view of the only sequence disagreement: an 84 bp intergenic region (olive box) present in the reference but not our assembly. The giant flagellin gene is represented by a magenta arrow. Other coding sequences are represented by yellow arrows.
