# Supplementary material for: Giant flagellins form thick flagellar filaments in two species of marine γ-proteobacteria
Source: PLoS One. 2018 Nov 21;13(11):e0206544. doi: 10.1371/journal.pone.0206544 (PMC6248924; doi:10.1371/journal.pone.0206544)
Supplement: S3 Fig — (A) SDS-PAGE gel of proteins isolated from the surface of B. marisrubri by acid depolymerisation, as described in materials and methods. The arrow indicates the 120 kDa band that was cut out and used for identification by trypsin digestion and mass spectrometry. (B) Summary of proteins identified by comparison of peptide fingerprints against a custom Mascot database. Protein score is -10 × Log(P), where P is the probability that the observed match is a random event. Protein scores outside of the green hatched area (>36) are significant (p<0.05). (PDF) [file pone.0206544.s011.pdf]

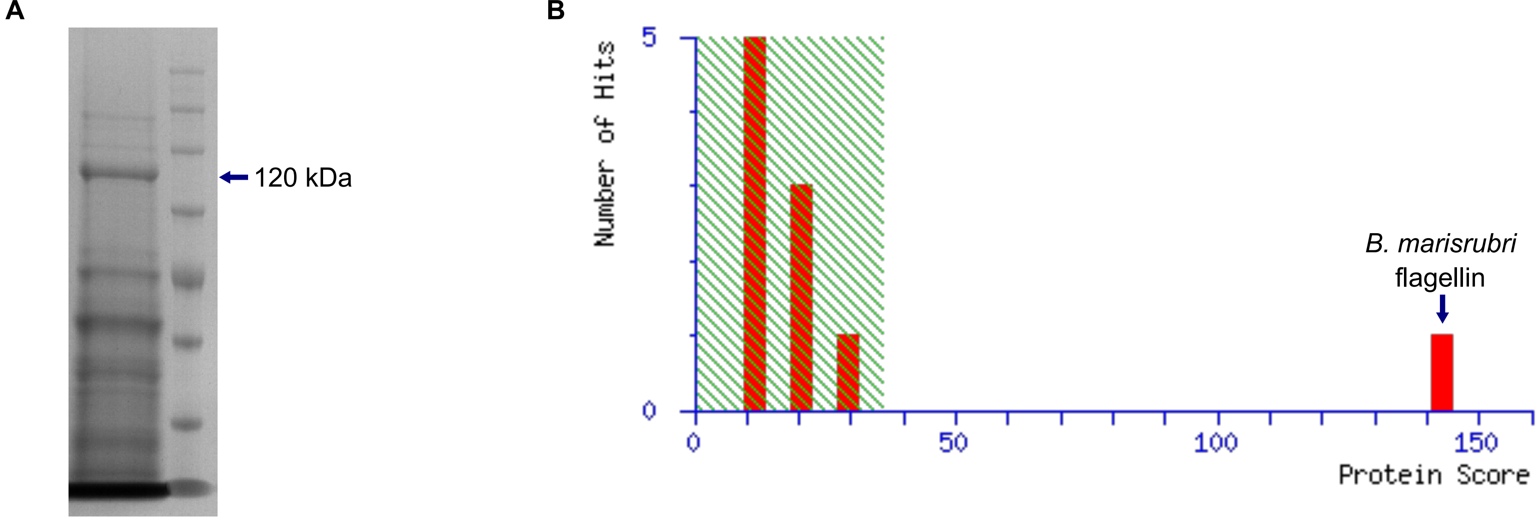

S3 Fig. Isolation and identification of the giant flagellin protein from *Bermanella marisrubri* Red 65. (A) SDS-PAGE gel of proteins isolated from the surface of *B. marisrubri* by acid depolymerisation, as described in materials and methods. The arrow indicates the 120 kDa band that was cut out and used for identification by trypsin digestion and mass spectrometry. (B) Summary of proteins identified by comparison of peptide fingerprints against a custom Mascot database. Protein score is -10 × Log(P), where P is the probability that the observed match is a random event. Protein scores outside of the green hatched area (>36) are significant ($p<0.05$).
